# Automatic epileptic seizure detection based on persistent homology

**DOI:** 10.3389/fphys.2023.1227952

**Published:** 2023-12-12

**Authors:** Ziyu Wang, Feifei Liu, Shuhua Shi, Shengxiang Xia, Fulai Peng, Lin Wang, Sen Ai, Zheng Xu

**Affiliations:** ^1^ School of Science, Shandong Jianzhu University, Jinan, China; ^2^ Medical Rehabilitation Research Center, Shandong Institute of Advanced Technology, Chinese Academy of Sciences, Jinan, China; ^3^ The Fifth People’s Hospital of Jinan, Jinan, China

**Keywords:** EEG, seizure detection, epilepsy, persistent homology, Vietoris-Rips complex

## Abstract

Epilepsy is a prevalent brain disease, which is quite difficult-to-treat or cure. This study developed a novel automatic seizure detection method based on the persistent homology method. In this study, a Vietoris–Rips (VR) complex filtration model was constructed based on the EEG data. And the persistent homology method was applied to calculate the VR complex filtration barcodes to describe the topological changes of EEG recordings. Afterward, the barcodes as the topological characteristics of EEG signals were fed into the GoogLeNet for classification. The persistent homology is applicable for multi-channel EEG data analysis, where the global topological information is calculated and the features are extracted by considering the multi-channel EEG data as a whole, without the multiple calculations or the post-stitching. Three databases were used to evaluate the proposed approach and the results showed that the approach had high performances in the epilepsy detection. The results obtained from the CHB-MIT Database recordings revealed that the proposed approach can achieve a segment-based averaged accuracy, sensitivity and specificity values of 97.05%, 96.71% and 97.38%, and achieve an event-based averaged sensitivity value of 100% with 1.22 s average detection latency. In addition, on the Siena Scalp Database, the proposed method yields averaged accuracy, sensitivity and specificity values of 96.42%, 95.23% and 97.6%. Multiple tasks of the Bonn Database also showed achieved accuracy of 99.55%, 98.63%, 98.28% and 97.68%, respectively. The experimental results on these three EEG databases illustrate the efficiency and robustness of our approach for automatic detection of epileptic seizure.

## 1 Introduction

Epilepsy is a chronic brain disease caused by the sudden abnormal discharge of brain neurons, and worldwide more than 70 million of people are suffering from epilepsy (Thijs et al., 2019; Bandopadhyay et al., 2021). When an epileptic occurs, the patients will lose consciousness, have convulsions, and cannot control their body movements. Epilepsy increases the risk of physical injuries, and accidents such as slipping and fainting caused by epilepsy would lead secondary injury to patients (Kaushik et al., 2022). Consequently, timely detection and intervention are crucial for patients.

Electroencephalogram (EEG) reflects the electrophysiological activities about brain nerve cells on the cerebral cortex or scalp, and records a lot of physiologic and pathological information. EEG is widely used in research in the field of brain science, such as disease detection (van der Zande et al., 2018; Oh et al., 2020), emotion recognition (Liu et al., 2020; Iyer et al., 2023), and sleep disorder diagnosis(Sharma et al., 2021; Chen et al., 2023) etc. The analysis of EEG signals can provide a diagnostic basis and an effective treatment for abnormal functioning of the brain, so studying EEG signals is a common way to analyze brain diseases (Acharya et al., 2013). Meanwhile, EEG is widely used for the epilepsy detection and treatment due to its low sampling cost and high time resolution (Vidyaratne and Iftekharuddin, 2017). However, the traditional epilepsy-detecting method requires the physicians to visually evaluate and manually label EEG signals, which is complicated and extremely expensive. Moreover, EEG recordings often last several hours, so it is actually a quite burdensome work for the physicians. Therefore, the automatic detection method of epileptic seizures has great significance to the long-term monitoring, diagnosis and treatment of epilepsy patients (Liu et al., 2012).

In recent years, many researchers have done a large number of works about the automatic epileptic seizure detection. And these studies are mainly divided into two directions: the feature extraction and classification (Vidyaratne and Iftekharuddin, 2017). About 90% studies focused on developing an effective method of feature extraction to find the most prominent EEG features that can be used for the epilepsy diagnosis (Hussein et al., 2018). Extracting features in the time domain, frequency domain, and time-frequency domains of EEG signals has always been a focus of past work, and these presented feature extraction methods were evaluated based on several open EEG databases (Alotaiby et al., 2014).

In 2011, D. Wang et al. applied the best basis-based wavelet packet entropy to extract feature parameters of EEG signals (Wang et al., 2011). In 2014, a framework based on the wavelet-based nonlinear features was proposed by Chen et al. (2014), which extracted some features on the selected single channel at the different resolutions of time and frequency. Zhang et al. (2017) explored a quadratic feature extraction method based on the Autoregression (AR) and Variational Mode Decomposition (VMD) for epilepsy detection in 2017. Bhattacharyya and Pachori (2017) proposed a novel method based on the Empirical Wavelet Transform (EWT), which was effective for epilepsy detection about long time EEG recordings on five channels. In 2018, Hussein et al. (2018) used a new epilepsy detecting algorithm based on the L1-Penalized Robust Regression (L1PRR). Their study showed that the algorithm could improve the detection accuracies and robustness for the EEG signals with artifacts and white noise (Hussein et al., 2018). Sharma et al. (2018) proposed that Iterative Filtering (IF) is suitable for analyzing non-stationary signals and better than traditional data adaptive analysis methods. In 2021, Mandhouj et al. (2021) used the Short-Time Fourier Transform (STFT) tool to do the time-frequency analysis of the EEG signal and create the EEG spectrum image, which reducing the complexity of the feature extraction process (Mandhouj et al., 2021). Moreover, A. Zarei and Asl (2021) introduced a new method for automatic detection of seizures based on the Discrete Wavelet Transform (DWT) and Orthogonal Matching Pursuit (OMP). They used the DWT and OMP to decompose EEG segments into different sub-bands, calculate features according to different coefficients, and used the Sequential Forward Feature Selection (SFFS) method to choose the best collection of features (Zarei and Asl, 2021). In 2022, Rajinikanth et al. (2022) proposed a method to use the Synchro Extracting Transform (SET) to examine EEG signals (Rajinikanth et al., 2022). A. Anuragi et al. proposed using the FBSE-EWT algorithm to extract sub-bands from EEG and reconstruct them into a 3D Phase-Space Representation (PSR). This method was suitable for analyzing non-stationary EEG signals and when using Extra Tree (ET) classifier, a classification accuracy of 100% can be achieved (Anuragi et al., 2022).

Machine learning is a mature technique. Many researchers used machine learning for epilepsy detection in a large number of studies (Vidyaratne and Iftekharuddin, 2017). A.H. Shoeb et al. put forward an automatic epilepsy detection method for the patients. This method used the Support Vector Machine (SVM) for feature classification, which can offer 96% prediction sensitivity (Shoeb and Guttag, 2010). Moreover, Billeci et al. (2018) introduced a patient-specific method for predicting epileptic seizures based on the combination of the Heart Rate Variability (HRV) parameters and Recurrence Quantification Analysis (RQA) parameters, and it incorporated EEG and ECG features, and fed them into the SVM for classification (Billeci et al., 2018). The research of Sanchez-Hernandez et al. (2022) showed that the performance of the classifier may be affected by many factors, and evaluated the combination of six feature selection methods and five classification models, and obtained the best combination (Sanchez-Hernandez et al., 2022). Deep learning algorithm is more effective in processing large and complex bioelectric signals, so it has been gradually utilized in the field of epilepsy detection in recent years, especially the Convolutional Neural Network (CNN) (Hu et al., 2019). S. Raghu et al. used the CNN and transfer learning to classify seven variants of seizures and non-seizure EEG. They used 10 pretrained networks to identify the best network for the proposed study. Then, they achieved higher classification accuracy of 82.85% using transfer learning, and the highest classification accuracy of 88.30% was achieved using the extract image feature approach (Raghu et al., 2020).

However, most of the previous researches selected a specific single channel from the multi-channel EEG signals, or separated the multi-channel, extracted the features separately and then spliced the features, and few studies analyzed the multi-channel EEG directly. The separation of signals and the features splicing need more calculating cost and time, and this would result in missing the best opportunity for disease treatment. Synchronization analysis of high-dimensional EEG signals is the key for timely automatic epileptic seizure detection. Persistent homology is a new method in topological data analysis. This method is applicable to high-dimensional data analysis and has the characteristics of not being limited by the threshold. It is a valid instrument for analyzing high-dimensional nonlinear data and nonlinear structure (Liang et al., 2021). Persistence was first proposed by H. Edelsrunner et al. (Edelsbrunner et al., 2002) in 2002, and it has been applied in many different research fields (Parks and Marchette, 2016). Lee et al. (2012) proposed a brain connectivity model using the persistent homology to avoid the single fixed threshold (Lee et al., 2012). Pachauri et al. (2011) explored the persistence of topological features induced by cortical thickness signals. The research results showed that persistent homology had the great advantage in capturing subtle anatomical variations in complex data (Pachauri et al., 2011). Persistent homology includes multiple complexes, of which the Vietoris-Rips (VR) complex is a practical complex suitable for analyzing high-dimensional data (Zomorodian, 2010). Guo et al. (2022) used persistent homology and VR complex to construct multi-scale brain network to extract features from the EEG signals of schizophrenic patients for analysis and demonstrated their stability as a biological reference standard. Fernández and Mateos (2022) used persistent homology to calculate topological biomarkers from the signals as features, and experiments have shown that biomarkers can effectively detect changes in brain dynamics (Fernández and Mateos, 2022). Yan et al. (2023) proposed an analysis method based on persistent homology for EEG emotion recognition, which extracts topological features from different EEG rhythm bands through VR filtering.

This study developed a new epileptic seizure detecting method based on the persistent homology, which can analyze the multi-dimensional EEG signals directly. First, the multi-dimensional EEG data are embedded into the metric space, to generate a point cloud matrix. Then, the VR complexes are born, growing, and disappearing gradually in the VR composite filtering processing. And, a persistence barcode is generated to record this process. Finally, GoogLeNet model is employed for the classification. This proposed new method can be directly applied to multi-channel EEG signals without separating channels, and is suitable for the epileptic detection. Three EEG epilepsy databases were applied to evaluate the performance of this method. The experimental results demonstrate that it has great performance, high reliability and robustness for the epileptic seizure detection.

The rest of the paper is organized as follows: Section 2 describes the EEG databases and proposed the epileptic seizure detection approach; Section 3 presents the experiments and results; Section 4 discuss and analyze the outcome of experiment. Finally, Section 5 concludes this work.

## 2 Materials and methods

**FIGURE 1:**
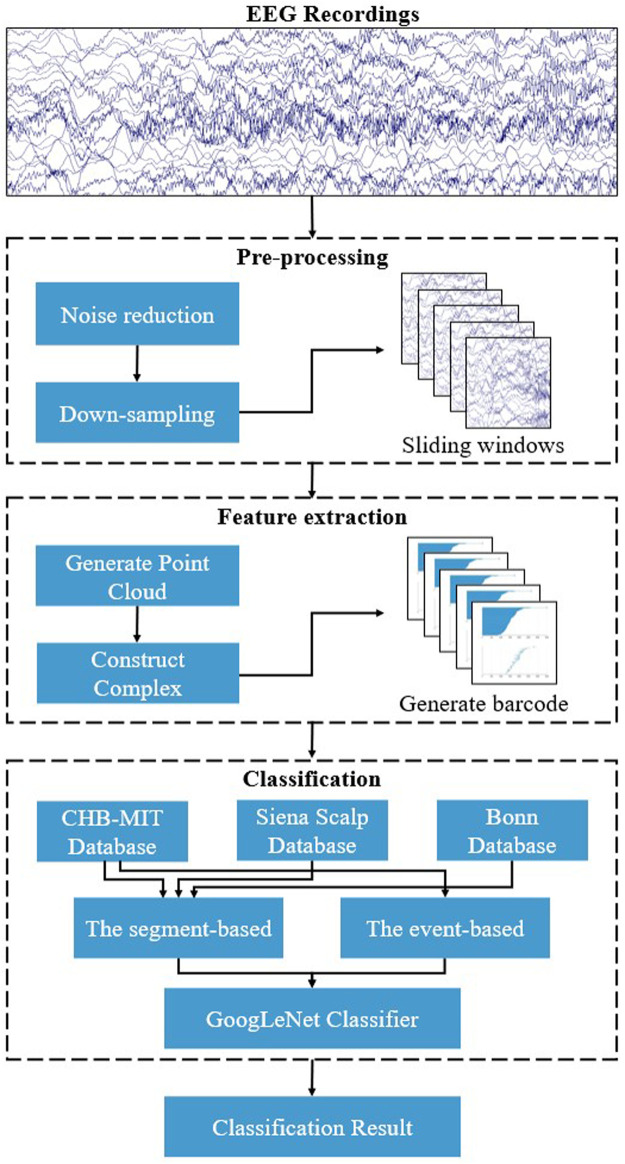
The main flowchart of the proposed approach for automatic seizure detection.

The flowchart of the proposed approach for automatic seizure detection is shown in Figure 1. At first, a pre-processing step is necessary, including the noise reduction to eliminate noise disturbances and down-sampling of the original EEG recordings to reduce computing costs, and the preprocessed signals are segmented into 2-s fragments. Then, the complex filtration model is constructed, and the topological features are extracted using persistent homology. Finally, the GoogLeNet is employed for classifying features.

### 2.1 EEG database

In this study, three EEG databases have been used to evaluate the performance of the proposed approach. These three databases are the CHB-MIT Scalp EEG Database, the Siena Scalp Database, and the Bonn Database.

#### 2.1.1 CHB-MIT Scalp EEG Database

The first database used in this study is the CHB-MIT Scalp EEG Database (Shoeb and Guttag, 2010), which contains 24 cases of scalp EEG recordings from 23 pediatric patients, including 5 male patients, 17 female patients, and one unspecified gender. The case “Chb24” is a case without age and gender, which was later added to the database. “Chb21” and “Chb01” were obtained from the same patient, “Chb21” is the EEG recording obtained for the second time after 1.5 years. Each recording is sampled at 256 Hz. Most recordings contain 23 EEG channels, which are based on the international 10–20 electrode placement system. All recordings totaled 872 h, including 198 seizures, with each recording containing between 0 and 5 epileptic seizures. More details of selected recordings from this database used in this study are described in Table 1. Since the channels and the order of the channels were different between different records, the channels are also selected to ensure that the channels remained consistent for each recording in this study (FP1-F7, F7-T7, T7-P7, P7-O1, FP1-F3, F3-C3, C3-P3, P3-O1, FP2-F4, F4-C4, C4-P4, P4-O2, FP2-F8, F8-T8, P8-O2, P7-T7, T7-FT9, FT9-FT10, FT10-T8).

**TABLE 1:** Details of selected recordings of the CHB-MIT Database and Siena Scalp Database.

CHB-MIT database					Siena Scalp Database				
Patient ID	Gender	Age	No. of seizures	Seizure minutes	Patient ID	Gender	Age	No. of seizures	Seizure minutes
Chb01	F	11	7	7:22	PN00	M	55	5	5:25
Chb02	M	11	3	2:52	PN01	M	46	2	2:08
Chb03	F	14	7	6:42	PN03	M	54	2	4:04
Chb04	M	22	4	6:18	PN05	F	51	3	1:44
Chb05	F	7	5	9:18	PN06	M	36	5	4:42
Chb06	F	1.5	10	2:33	PN07	F	20	1	1:02
Chb07	F	14.5	3	5:25	PN09	F	27	3	3:23
Chb08	M	3.5	5	15:19	PN10	M	25	10	6:05
Chb09	F	10	4	4:36	PN11	F	58	1	0:55
Chb10	M	3	7	7:27	PN12	M	71	4	4:50
Chb11	F	12	3	13:26	PN13	F	34	3	4:24
Chb12	F	2	27	16:29	PN14	M	49	4	2:43
Chb13	F	3	10	7:20	PN16	F	41	2	3:50
Chb14	F	9	8	2:49	PN17	M	42	2	2:33
Chb15	M	16	20	32:02					
Chb16	F	7	8	1:09					
Chb17	F	12	3	4:53					
Chb18	F	18	6	5:17					
Chb19	F	19	3	3:56					
Chb20	F	6	8	4:54					
Chb21	F	13	4	3:19					
Chb22	F	9	3	3:24					
Chb23	F	6	7	7:04					
Chb24	-	-	16	8:31					

#### 2.1.2 Siena Scalp Database

Moreover, in order to ensure the robustness of the proposed approach, another EEG database have been utilized, Siena Scalp Database (Detti et al., 2020). This database was acquired by the Unit of Neurology and Neurophysiology at the University of Siena, Italy, and it contains 41 scalp EEG recordings from 14 patients, including 9 male and 5 female patients, respectively. The sampling rate is 512 Hz. All recordings totaled 128 h, including 47 seizures, with each recording containing between 1 and 3 epileptic seizures. More details overviews of selected recordings of this database are summarized in Table 1.

#### 2.1.3 Bonn Database

Furthermore, to evaluate the applicability of the proposed approach on single-channel EEG recordings and multiclassification, the Bonn Database has also been used in this study. This database was collected by the Department of Epileptology at University of Bonn, Germany (Andrzejak et al., 2001). The database consists of five sets (from Set A to Set E) of single-channel EEG recordings, and each set contains 100 segments with a length of 23.6 s, which are sampled at 173.61 Hz and based on the international 10–20 system. Sets A and B were collected from five healthy subjects. Set A consisted of subjects with eyes open, Set B has consisted of subjects with eyes closed. Sets C, D and E were collected from five patients with epilepsy. While Sets C and D only include interictal recording. The recordings in Set D were collected from within the epileptogenic zone of the brain, and the recordings in Set C were collected from the formation of the opposite hemisphere. The recordings in Set E are all with epileptic seizures. This database has been filtered at frequencies between 0.53 and 40 Hz.

### 2.2 Pre-processing

Several recordings in the CHB-MIT Database contain different channels when compared with others, so it is necessary to choose the same channels to ensure the uniformity of all recordings. But the channels contained from each subject in the Siena Scalp Database varied widely, which made it infeasible to standardize them in this study. EEG generated cortically is susceptible to contamination by non-cerebral artifact origins such as blinking, ocular movements, and electromyography (EMG). Moreover, it is also contaminated by external electromagnetic activity such as the electrical disturbances and instrument noise, all of which would diminish the quality of EEG recordings and cause the wrong features to be extracted (Upadhyay et al., 2016). Therefore, the noise reduction is necessary for the EEG recordings. Since most seizure activity occurs in this 0.5–40 Hz frequency range (Zarei and Asl, 2021), band-pass filtering of 0.5–40 Hz is carried out in this study for the two databases mentioned above. And down-sampled the sampling frequency of the Siena Scalp Database to 256 Hz. Bonn Database has already been pre-processed, so this pre-processing step is not necessary.

The sliding windows was used to segment the seizure period into 2-s fragments $E_{l}$ . The same operation is carried out on the interictal period. There are equal numbers of the seizure and interictal fragments.

### 2.3 Persistent homology

Persistent homology is a basic tool in Topological data analysis. It has applications in various domains such as image recognition, biology, chemistry, and network analysis (Fugacci et al., 2016). Persistent homology describes the changes in homology that occur to the complex in Topological space.

#### 2.3.1 Persistent homology

**FIGURE 2:**
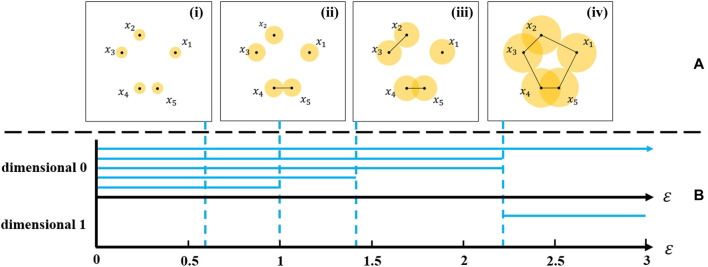
**(A)** Construction of Vietoris–Rips complex. **(i–iv)** are VR(X,ε) complexes of the point cloud data with ε = 0.6, 1.0, 1.44, and 2.35 respectively. **(B)** The persistence barcode generated by this VR complex.

Let $\left(X,d\right)$ be a metric space, and a Vietoris-Rips (VR) complex with *ε* parameters is a simple complex denoted by $VR\left(X,\varepsilon \right)$ , and whose vertex is the set *X.* Subset $\left\{x_{0},x_{1},\cdots ,x_{k}\right\}$ of *X* generates a *k*-complex if and only if $d\left(x_{i},x_{j}\right)\leq \varepsilon$ for all 0≤i,j≤k . However, there is usually a middle range, where $VR\left(X,\varepsilon \right)$ has homology groups isomorphic to those of *X*, and therefore has Betti numbers equal to those of *X.* Edelsbrunner et al. (2002) have made the following observation: whenever $\varepsilon \leq \varepsilon^{\prime }$ , there exists a natural inclusion of simplicial complexes $VR\left(X,\varepsilon \right)↪VR\left(X,\varepsilon^{\prime }\right)$ . From this functoriality property, one gets a linear transformation $H_{k}\left(VR\left(X,\varepsilon \right)\right)\to H_{k}\left(VR\left(X,\varepsilon^{\prime }\right)\right)$ for any *k*. In order to study the homology of a space using a point cloud sampled from it, one should keep track of all the linear transformations described above and the whole system of vector spaces $H_{k}\left(VR\left(X,\varepsilon \right)\right)$ . This system is called persistent Vector space (Zomorodian and Carlsson, 2005). For each persistence vector space, there is an invariant called barcode, which is a finite collection of intervals. Intuitively, persistent barcodes are a set of intervals that represent the life of nontrivial circles during the process of complex growth. The left endpoint of the interval represents the birth of new topological properties, while the right endpoint of the interval represents its death. Longer intervals are considered to represent inherent characteristics of the complex, while shorter intervals are considered noise or improper sampling. Figure 2B gives an example of a barcode. Persistent homology is an effective tool to describe the change process of topological properties of the simple complexes.

VR complexes constructed from a point cloud data and how to generate a barcode are described in the following example to help understand persistent homology. Constructed of the VR complexes in the example is shown in Figure 2. Place 5 points on the plane [Coordinates of points are (2,2), (0,3), (−1,2), (0,0), (1,0)], and draw five circles centered around the points with the radius ε/2 . Suppose that $\beta_{0}$ is the 0-dimensional Betti number, represents the number of components, and $\beta_{1}$ is the 1-dimensional Betti number, represents the number of loops. When ε<1.0 , the five points are independent of each other and each point is an independent component. Such as in Figure 2Ai, when ε=0.6 , there are five components and no one loop formed. Therefore, the 0-dimensional Betti number $\beta_{0}=5$ and the 1-dimensional Betti number $\beta_{1}=0$ . In Figure 2Aii, along with ε increase to ε=1.0 , points $x_{4}$ and $x_{5}$ are connected, two points form one 1-dimensional simplex. There are four components, that is $\beta_{0}=4$ . In Figure 2Aiii, when ε=1.44 , the two upper left points $x_{2}$ and $x_{3}$ are connected form a new component, this means $\beta_{0}=3$ . In Figure 2Aiv, a loop is formed, so $\beta_{0}=1$ and $\beta_{1}=1$ when ε=2.35 . Then, the persistence barcode calculated based on persistent homology is shown in the Figure 2B.

#### 2.3.2 Vietoris–Rips complex filtration model

For generating the point cloud, each EEG segment with a duration of 2 s is segmented into *T*-s epochs using a sliding window. The segmented fragments are folded and arranged sequentially, such that the number of points in the point cloud can be increased. For an EEG with n-channels, a point cloud with 2*n* points can be obtained by folding once, and so on. Let each point in the constructed point cloud be the point *X* in Euclidean space, with threshold *ε* representing the Euclidean distance between point clouds.

From this, a VR filtration model of the EEG can be defined as:

$$VR\left(X,\varepsilon_{1}\right)\subseteq VR\left(X,\varepsilon_{2}\right)\subseteq \cdots \subseteq VR\left(X,\varepsilon_{M}\right)$$

**FIGURE 3:**
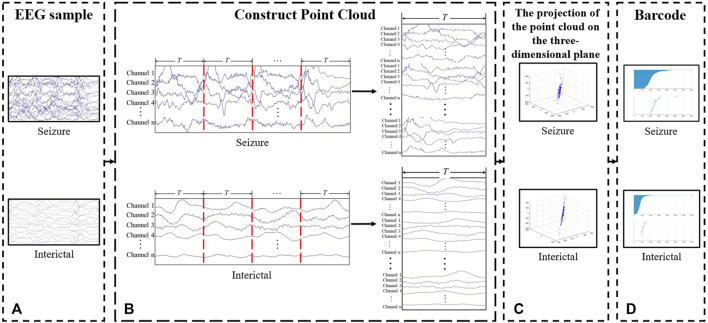
Process of feature extraction. **(A)** EEG fragment. **(B)** Construct point cloud. Divide and fold the EEG fragments to increase the number of the points in the point clouds. **(C)** Point cloud. The figure shows the projection of the point cloud on the three-dimensional plane. **(D)** Barcode.

As mentioned in Section 2.3.1, this process can be intuitively felt through the VR filtration barcode. The horizontal lines in the barcode represent the topological properties, with the left end of the line represents the appeal of a new topological property, and the right end represents it disappear. The change rules of topological property of an EEG can be described through Betti number. $\beta_{0}$ is the 0-dimensional Betti number used to represents the number of components of the EEG recordings, and the number of 1-dimensional holes is represented by the 1-dimensional Betti number $\beta_{1}$ . Figure 3 shows the construction process of a VR complex filtration model in the proposed approach.

### 2.4 Classifier

In the classification step, the GoogLeNet classifier was used for the classification of seizure and interictal samples. GoogLeNet is a new deep learning algorithm proposed by Szegedy et al. (2015). In contrast to the traditional linear convolutional structure of CNNs, which contains a nonlinear convolution structure, namely, Inception (Szegedy et al., 2015). The inception enables a greater retention of input information and solves many negative effects due to the increased depth of the network. The two main advantages of Inception are using 1 × 1 convolution for up- or down-dimensioning, and performing simultaneous convolutional re-aggregation at multiple sizes, which can reduce the training parameters of the model and the total calculation.

### 2.5 Experimental design

Two classification experiments were designed in this study to test the classification performance of our proposed method: segment-based classification (Experiment 1); and event-based classification (Experiment 2). In Experiment 1, the fragments of each database were divided into 70% and 30% for training and testing respectively. This experiment was used to calculate the accuracy of classification. In Experiment 2, the number of epileptic seizure events for each subject in the CHB-MIT Database was noted as *S*. The classifier was trained using EEG samples of all interictal samples and *S*-1 seizure events, and the testing was done on the remaining 1 seizure event. This step was repeated *S* times, so that each seizure event is tested once. This was used to calculate the detection latency and the number of error detections of epilepsy.

### 2.6 Evaluation method

The evaluation indicators of model used in this study are Accuracy (*Acc*), Sensitivity (*Sens*), Specificity (*Spec*), Precision (*Pre*), modified Accuracy (*mAcc*), and comprehensive index *F1* measure, which are defined as follows:

$$Acc=\frac{TP+TN}{TP+FP+TN+FN}\times 100$$

$$Sens=\frac{TP}{TP+FN}\times 100$$

$$Spes=\frac{TN}{TN+FP}\times 100$$

$$Pre=\frac{TP}{TP+FP}\times 100$$

$$F1=\frac{TP}{TP+0.5\left(FP+FN\right)}\times 100$$

$$mAcc=\frac{Sens+Spec}{2}$$

where, the True Positive (*TP*) represents the number of seizure fragments correctly classified; the False Positive (*FP*) is the number of interictal fragments incorrectly classified to be the seizure fragments; the True Negative (*TN*) the number of correctly classified the interictal fragments; the False Negative (*FN*) is the number of seizure fragments incorrectly classified as the interictal indeed.

## 3 Results

The proposed approach was evaluated on three public EEG databases, and the results are shown in this section.

### 3.1 Detection results of CHB-MIT Database

#### 3.1.1 Detection results at the segment-based

The detecting results at the segment-based of the CHB-MIT Database are shown in Table 2. It can be seen from this table, the proposed approach provides a mean accuracy of 97.05%, a mean sensitivity of 96.71%, and a mean specificity of 97.38%. Moreover, the precision, modified accuracy and *F*1 measure are also shown, 97.36%, 97.02%, and 97.05%, respectively. The subject “Chb06,” “Chb12,” “Chb15,” and “Chb24” had low performance in all evaluation indexes, of which “Chb12” was the lowest. However, the evaluation indexes of 18 subjects are close to or more than 95%, accounting for 3/4 of all subjects.

**TABLE 2:** The detection results of the proposed approach at the segment-based.

Patient ID	*Acc*%	*Sens*%	*Spec*%	*Pre*%	*F*1%	*mAcc*%
Chb01	100	100	100	100	100	100
Chb02	98.04	96.08	100	100	98	98.04
Chb03	99.58	100	99.15	99.16	99.57	99.58
Chb04	100	100	100	100	100	100
Chb05	97.89	97.59	98.19	98.18	97.89	97.89
Chb06	90.7	93.02	88.37	88.89	90.91	90.7
Chb07	100	100	100	100	100	100
Chb08	99.45	100	98.91	98.92	99.46	99.45
Chb09	98.78	97.56	100	100	98.77	98.78
Chb10	99.24	98.48	100	100	99.24	99.24
Chb11	99.59	99.17	100	100	99.58	99.59
Chb12	88.93	86.85	91	90.61	88.69	88.93
Chb13	92.25	89.15	95.35	95.04	92	92.25
Chb14	93.75	95.83	91.67	92	93.88	93.75
Chb15	89.19	88.68	89.7	89.59	89.13	89.19
Chb16	100	100	100	100	100	100
Chb17	100	100	100	100	100	100
Chb18	96.24	94.62	97.85	97.78	96.17	96.24
Chb19	100	100	100	100	100	100
Chb20	98.84	98.84	98.84	98.84	98.84	98.84
Chb21	98.28	100	96.55	96.67	98.31	98.28
Chb22	100	100	100	100	100	100
Chb23	99.2	100	98.4	98.43	99.21	99.2
Chb24	89.19	85.14	93.24	92.65	88.73	89.19
**Average**	**97.05** ± **3.89**	**96.71** ± **4.59**	**97.38** ± **3.65**	**97.36** ± **3.66**	**97.02** ± **3.95**	**97.05** ± **3.89**

Bold values are used to distinguish between individual values and mean values.

#### 3.1.2 Detection results at the event-based

Experiment 2, the event-based classification, was also conducted on the CHB-MIT Database. All 181 seizure events of 24 subjects were detected by the proposed approach successfully. This corresponds to a detection sensitivity of 100%. Table 3 summarizes the results at the event-based. And, Table 3 also records the average, lowest, and highest detection seizure detection latencies for each subject. The proposed approach achieves a mean seizure detection latency of 1.22 s. And, 73% of seizures being detected without the detection latency and 95% of all seizures were detected with less than or equal to 5 s after the onset. The minimum latency achieved for each subject was 0 s except for “Chb15,” “Chb18,” “Chb22” and “Chb24” which showed minimum latency of more than 10 s “Chb24” has average detection latency is 4.125 s, while 96% of subjects’ average detection latencies are less than or equal to 4 s.

**TABLE 3:** The detection results of the proposed approach at the event-based.

Patient ID	No. of marked seizures	No. of true detections	*Sens*%	Lowest latency(s)	Highest latency (s)	Avg. latency (s)
Chb01	7	7	100	0	0	0
Chb02	3	3	100	0	0	0
Chb03	7	7	100	0	1	0.14
Chb04	4	4	100	0	2	0.5
Chb05	5	5	100	0	3	1
Chb06	10	10	100	0	4	0.9
Chb07	3	3	100	0	0	0
Chb08	5	5	100	0	0	0
Chb09	4	4	100	0	0	0
Chb10	7	7	100	0	0	0
Chb11	3	3	100	0	0	0
Chb12	27	27	100	0	4	0.88
Chb13	10	10	100	0	3	0.8
Chb14	8	8	100	0	1	0.25
Chb15	20	20	100	0	13	3.6
Chb16	8	8	100	0	1	0.125
Chb17	3	3	100	0	0	0
Chb18	6	6	100	0	16	2.66
Chb19	3	3	100	0	0	0
Chb20	8	8	100	0	2	0.25
Chb21	4	4	100	0	0	0
Chb22	3	3	100	0	12	4
Chb23	7	7	100	0	0	0
Chb24	16	16	100	0	48	4.125
**Total**	**181**	**181**	**100(mean)**	**0(mean)**	**4.58(mean)**	**1.22(mean)**

Bold values are used to distinguish between individual values and mean values.

### 3.2 Detection results of Siena Scalp Database

Moreover, the detection evaluation indexes of the Siena Scalp Database are shown in Table 4. It can be seen from Table 4 that the method in this study provides a mean accuracy of 96.41%, a mean sensitivity of 95.23%, and a mean specificity of 97.6%. Noteworthy, all subjects have the mean accuracy of more than 90%, and the evaluation indexes of nine subjects are close to or more than 95%, accounting for 64.3% of all subjects. The worst result, an accuracy of 90% with neither the sensitivity nor the *F*1 measure reached 90%, is acquired from the subject “PN10.”

**TABLE 4:** The detection results of the proposed approach for the Siena Scalp Database.

Patient ID	*Acc*%	*Sens*%	*Spec*%	*Pre*%	*F*1%	*mAcc*%
PN00	93.88	95.92	91.84	92.16	94	93.88
PN01	97.37	94.74	100	100	97.3	97.37
PN03	96.58	94.52	98.63	98.57	96.5	96.58
PN05	100	100	100	100	100	100
PN06	96.47	95.29	97.65	97.59	96.43	96.47
PN07	100	100	100	100	100	100
PN09	100	100	100	100	100	100
PN10	90	84.55	95.45	94.9	89.42	90
PN11	100	100	100	100	100	100
PN12	94.25	91.95	96.55	96.39	94.12	94.25
PN13	97.47	97.47	97.47	97.47	97.47	97.47
PN14	93.24	91.89	94.59	94.44	93.15	93.24
PN16	94.93	95.65	94.2	94.29	94.96	94.93
PN17	95.65	91.3	100	100	95.45	95.65
**Average**	**96.41** ± **2.91**	**95.23** ± **4.22**	**97.6** ± **2.61**	**97.56** ± **2.6**	**96.34** ± **3.01**	**96.42** ± **2.91**

Bold values are used to distinguish between individual values and mean values.

### 3.3 Detection results of Bonn Database

Similarly, using five sets from the Bonn Database to evaluate the classification performance of the proposed approach on different types of classification tasks, the results are shown in Table 5. The objects of Case 1 are Set A and Set E, and the objects of Case 3 are Sets A, B and Set E. The purpose of both cases is to detect healthy EEG recordings and seizure EEG recordings. The objects of Case 2 are Set C and Set E, and the objects of Case 4 are Set C, D and Set E. The purpose is to detect the EEG recordings in the interictal and seizure recordings.

**TABLE 5:** The detection results of the proposed approach for the Bonn Database.

Case	Tasks	*Acc*%	*Sens*%	*Spec*%	*Pre*%	*F*1%	*mAcc*%
1	A-E	99.55	100	99.09	99.09	99.55	99.55
2	C-E	98.63	99.7	97.58	98.64	98.65	98.64
3	AB-E	98.28	96.82	99.02	98	97.41	97.92
4	CD-E	97.68	98.79	97.12	94.49	96.59	97.95

The results shows that the detection of interictal and seizure (Case 2) could achieve an accuracy of 98.63% and a sensitivity of 99.69%. In addition, the results of the detection of healthy EEG recordings and seizure EEG recordings (Case 1) could achieve 99.55% accuracy and 100% sensitivity. This demonstrates that the approach in this study can also detect healthy EEG recordings from seizure in addition to interictal and seizure recordings. Moreover, the results also show that the approach is applicable to single-channel EEG and can achieve better results.

## 4 Discussions

This study constructed a new automatic seizure detection method based on the persistent homology, and it provides the satisfactory performance in all conducted experiments. Therefore, this section shows the comparison between the results of the proposed approach and the existing approaches in recent years. Furthermore, this study also analyzes the influences of different model constructions on the classification performance and the high detection latency appearing in the experiment, and discusses the performance of the approach on the multivariate classification.

### 4.1 Comparison with other research results

In this section, we compare the performances of the present study with those obtained in previous literatures. And we compare the detection results on the same database.

Table 6 summarizes the detailed comparison of the detection results between proposed methods with previous works on the CHB-MIT Database. From the segment-based detection results, our approach obtained a mean accuracy of 97.05%, comparable the results of the study by Zarei and Asl (2021), slightly lower than that reported by Li et al. (2021), significantly higher than other research results in Table 6. However, a total of 181 seizure events were used in this study involving all subjects, while the 131 seizure events used by C. Li et al. And the reliability and applicability of the proposed approach is better demonstrated by using as many seizure recordings from the database as possible. And the 100% event-based detection sensitivity is obviously higher than the other seizure detection approaches in Table 6. In addition, the average latency in this study is 1.22 s, which is better than 1.89 s in the study by Vidyaratne and Iftekharuddin (2017) and 4.6 s in the study by Shoeb and Guttag (2010).

**TABLE 6:** Comparison of detection results between the proposed approach and previous works on the CHB-MIT Database.

Authors	Method	Classifier			Patients		No. of seizures		Avg. *Acc*%	Avg. *Sens*%	Avg. *Spec*%	Event. *Sens*%	Avg. Latency (s)
Shoeb and Guttag (2010)	Energy of filter bank	SVM			24		163		NR	NR	NR	96	4.6
Kiranyaz et al. (2014)	Multi-dimensional particle swarm optimization	CNBC			21		NR		NR	89.01	94.71	NR	NR
Chen et al. (2014)	Wavelet-based nonlinear features	ELM			12		68		NR	92.6	NR	NR	NR
Fergus et al. (2015)	Feature-ranking algorithms, Principle component analysis	KNNC			24		171		NR	93	94	NR	NR
Vidyaratne and Iftekharuddin (2017)	Harmonic wavelet packet transform, Fractal dimension	RVM			22		150		NR	NR	NR	96	1.89
Yuan et al. (2018)	Earth mover’s distance	BLDA			24		145		95.74	95.65	95.75	94.48	NR
Kaleem et al. (2018)	Empirical mode decomposition-based dictionary approach	SVM			23		NR		92.91	94.27	91.55	NR	NR
Zabihi et al. (2020)	Nonlinear dynamics, nullclines	ANN			24		NR		95.11	91.15	95.16	NR	NR
Zarei and Asl (2021)	Orthogonal matching pursuit	SVM			23		182		96.04	95.96	96.39	NR	NR
Zarei and Asl (2021)	Discrete wavelet transform	SVM			23		182		97.09	96.81	97.26	NR	NR
Li et al. (2021)	Empirical mode decomposition, common spatial pattern	SVM			24		131		97.49	97.34	97.50	98.47	NR
**This work**	**Persistent Homology**	**GoogLeNet**			**24**		**181**		**97.05**	**96.71**	**97.38**	**100**	**1.22**

NR, not reported.

Bold values are used to distinguish our research results from those of others in the past.

Moreover, the results on the Siena Scalp Database are shown in Table 7. Compared with the results from the reference (Sanchez-Hernandez et al., 2022), both approaches have similar mean accuracy, but the approach in this study provides a mean sensitivity of 94.9%, which is better than 84%. Xiong et al. (2022) used RF to classify, and obtained a mean accuracy of 98.88%. However, they segmented the seizure period into 4-s fragments, while we segmented into 2-s. Jiang et al. (2023) proposed a seizure detection algorithm using an improved functional brain network structure for the feature extraction, and compared with their results, the proposed method in this study has better accuracy and specificity. These experimental results demonstrated the superiority of the seizure detection approach proposed in this study. The method proposed by Zhao et al. (2023) obtained an accuracy of 97.87% and a sensitivity of 97.4%, but the specificity of 96.99% was slightly lower than our method.

**TABLE 7:** Comparison of detection results between the proposed approach and previous works for the Siena Scalp Database.

Authors	Method	Classifier	Avg. Acc%	Avg. Sens%	Avg. Spec%
Sanchez-Hernandez et al. (2022)	Multiple feature extraction	KNN/SVM	96	84	NR
Xiong et al. (2022)	Improved genetic algorithm	RF	98.88	98.36	99.13
Jiang et al. (2023)	Pearson correlation coefficient and mutual information combined functional brain networks	SVM	95.68	97.72	93.9
Zhao et al. (2023)	Feature coupling block	CNN + Transformer	97.87	97.4	96.99
**This work**	**Persistent Homology**	**GoogLeNet**	**96.42**	**95.23**	**97.6**

NR, not reported.

Bold values are used to distinguish our research results from those of others in the past.

Furthermore, the comparison of the proposed approach as well as previous studies on the Bonn Database are shown in Table 8. In Case 1 (Sets A and E), the results of the proposed study is the same as those of the study (Yavuz et al., 2018), slightly lower than study (Nabil et al., 2020), and better than other previous works. In Case 2 (Sets C and E), the sensitivity of 99.7% in this study is better than that Samiee et al. (2015), the accuracy was similar to Aayesha et al. (2021) and higher than others. In Case 3 (Sets A, B and E), Anuragi et al. (2022) got the best classification performance, but different from us, they took 500 classification sample, while we took 2-s fragments as the sample, so we have 11,000 samples. In Case 2 and Case 4 (Sets C, D and E), the best results of accuracy and specificity are obtained by Yavuz et al. (2018), but the sensitivity of this study is quite better than other previous works.

**TABLE 8:** Comparison of detection results between the proposed approach and previous works for the Bonn Database.

Case	Tasks	Authors	Avg. *Acc*%	Avg. *Sens*%	Avg. *Spec*%
1	A-E	Wang et al. (2011)	99.449	NR	NR
		Kaya et al. (2014)	98	99	97
		Bhardwaj et al. (2016)	99.11	95.29	100
		Yavuz et al. (2018)	99.5	99	100
		Gupta et al. (2018)	94.85	95.4	94.3
		Nabil et al. (2020)	100	100	100
		Rajinikanth et al. (2022)	98.5	99	98
		**This work**	**99.55**	**100**	**99.09**
2	C-E	Siuly et al. (2011)	96.4	98	94.8
		Samiee et al. (2015)	98.5	99.3	97.7
		Yavuz et al. (2018)	99	98	100
		Gupta et al. (2018)	97.5	98	97
		Nabil et al. (2020)	95	96	94
		Aayesha et al. (2021)	99.81	99.62	100
		**This work**	**98.64**	**99.7**	**97.58**
3	AB-E	Yavuz et al. (2018)	99.67	99	100
		Gupta et al. (2018)	97.27	97.4	97.15
		Nabil et al. (2020)	2020	99	100
		Dash and Kolekar (2020)	2020	99.58	NR
		Anuragi et al. (2022)	2022	100	100
		**This work**	**98.28**	**96.82**	**99.02**
4	CD-E	Yavuz et al. (2018)	98.33	96	99.5
		Gupta et al. (2018)	96.92	96.85	97
		Nabil et al. (2020)	95.67	98	94.5
		Dash and Kolekar (2020)	97.5	NR	NR
		Aayesha et al. (2021)	98.89	98.48	99.08
		**This work**	**97.68**	**98.79**	**97.12**

NR, not reported.

Bold values are used to distinguish our research results from those of others in the past.

### 4.2 Applied to multivariate classification

**FIGURE 4:**
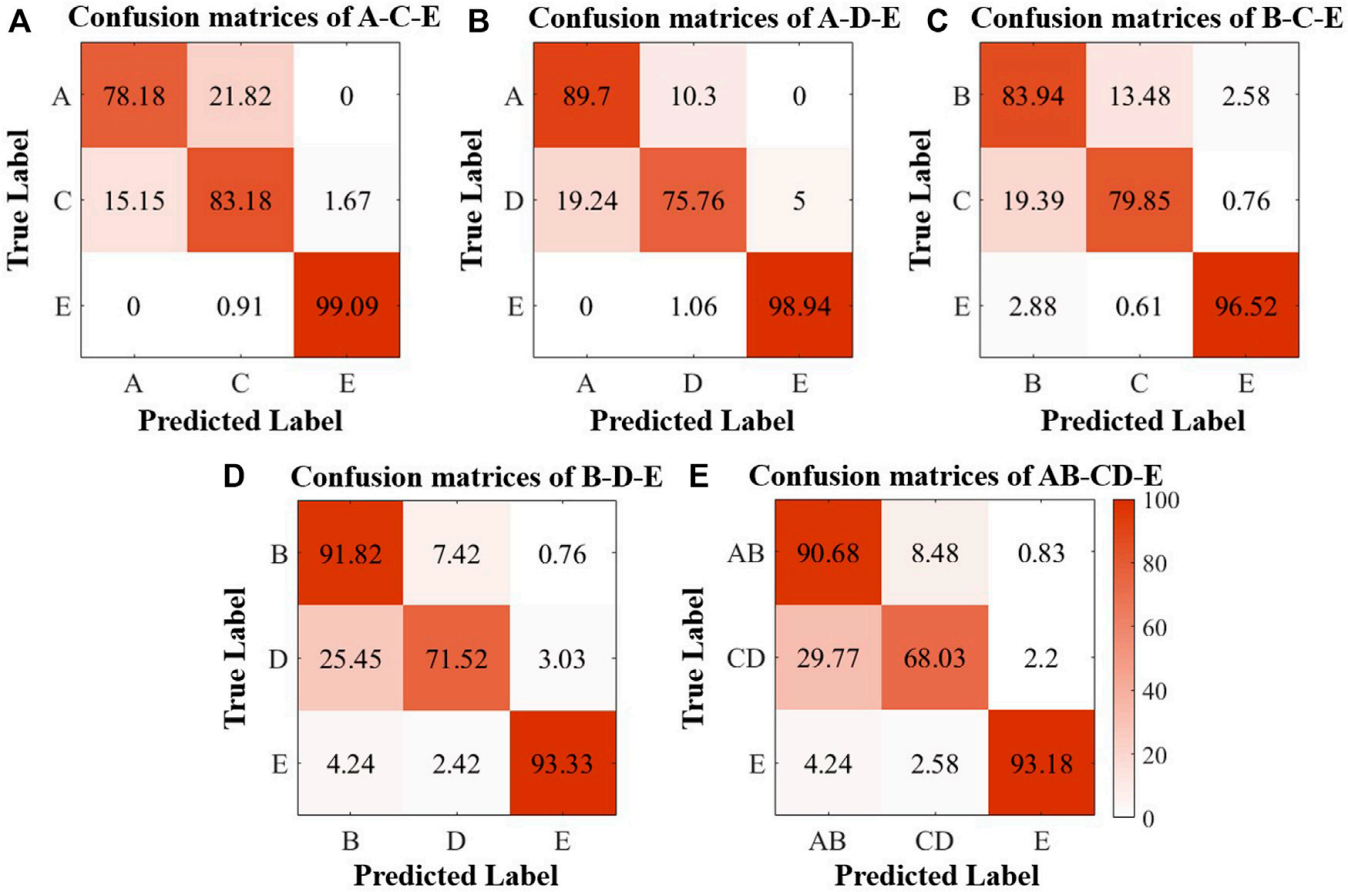
Confusion matrices of the classification results of the Bonn Database. **(A)** A-C-E; **(B)** A-D-E; **(C)** B-C-E; **(D)** B-D-E; **(E)** AB-CD-E.

In this study, we performed multiple classification tasks on several sets of the Bonn Database to test the feasibility of the proposed method for multiple EEG signals classification. Divides the five sets of the database into three categories, the healthy EEG recordings (Set A and Set B), the interictal EEG recordings of epileptic patients (Set C and Set D) and the seizure EEG recordings of epileptic patients (Set E), respectively. Carry out five different task combinations of the above three categories, and the classification results are shown in Figure 4.

As Figure 4 shows, the classification precision for seizures is still maintained at a high level in the five tasks, and the classification precision for healthy EEG recordings can achieve more than 80%, but most of the misclassification that occurred are concentrated in classifying healthy and interictal EEG recordings. Even so, the available multiclassification results still can demonstrate the feasibility of persistent homology in the multiple classifications of epilepsy.

### 4.3 Effects of different model constructions

**FIGURE 5:**
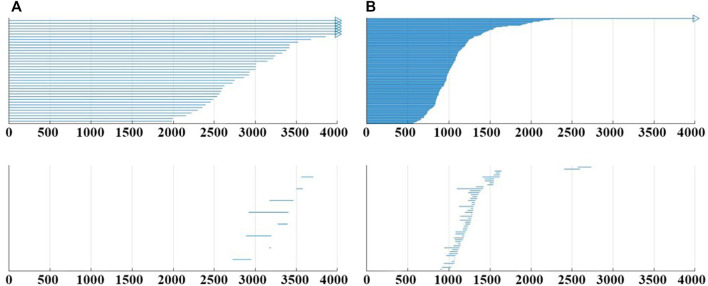
**(A)** Example of barcode when the point cloud with 38 points. **(B)** Example of barcode when the point cloud with 152 points. All them from subject CHB05_06, the 476–478 s.

Generally, the construction of VR complex filtration model will have direct influence on the detection results, which is discussed in this section. For a 2 s EEG segment, the original data is a fragment of *n* × 256 Hz × 2 s recording (where *n* is the number of channels). Without changing the original EEG data, the number of points in the point cloud can be increased by collapsing the channels to construct multiple VR complex filtration model. The results of construction will be visually represented in the barcode. Decompose the 2s EEG segment into two equal-length (1 s) segments, then overlap the two segments, and a data segment of 2*n* × 256 is obtained. And the VR complex filtration model and barcode from the point cloud with 2*n* points are constructed. Similarly, using the same method, the complex filtration model from the point cloud with 4*n*, and 8*n* points can also be constructed, and the corresponding barcode can be generated. In the study, the 19-channel from the CHB-MIT database was used so that the barcodes could be obtained from the point cloud with 19, 38, 76, and 152 points, respectively. As shown in Figure 5, for the same segment of the EEG recording with different filtration model constructions, the changes in the barcode are not only reflected in the increase of features in the 0-dimensional, but the changes are more obvious in the 1-dimensional.

**FIGURE 6:**
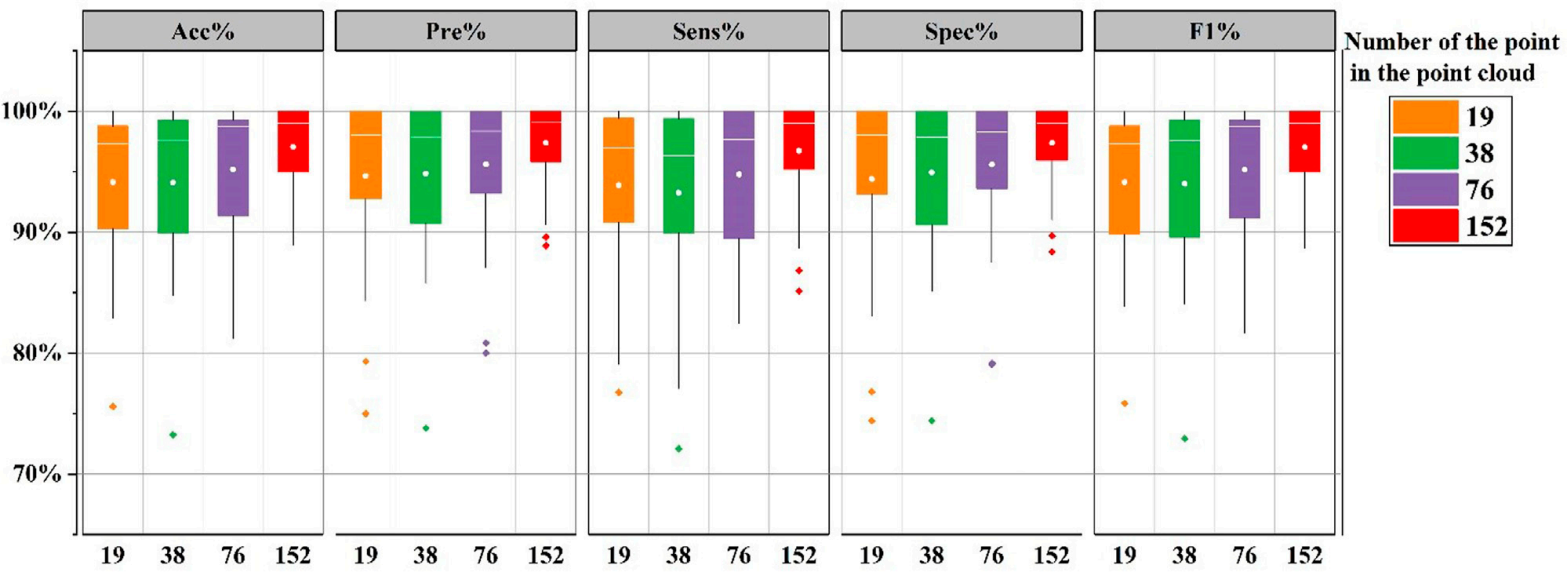
The boxplot of evaluation indices. Comparison of detection results by different model constructions. Among the 5 constructions, the number of points in the point cloud are 19, 38, 76, and 152, respectively.

Figure 6 shows the evaluation indexes for the 24 subjects in the CHB-MIT database under the five different model constructs. When the number of points is 152, the proposed approach provides the mean accuracy 97.05%. Overall, as the number of points in the point cloud increases, all evaluation indexes improve to different degrees and the best indexes are gained at 152. Continuing to increase the number of point cloud points has been experimented in this work. But it bears a burden of large computation and the computation time. The results proved that although the number of points had an influence on the evaluation indexes, the overall was relatively stable, and all the indexes could be maintained above 93%, which reflects that the proposed method has the high robustness and stability.

### 4.4 Analysis of high detection latency and real-time validation

**FIGURE 7:**
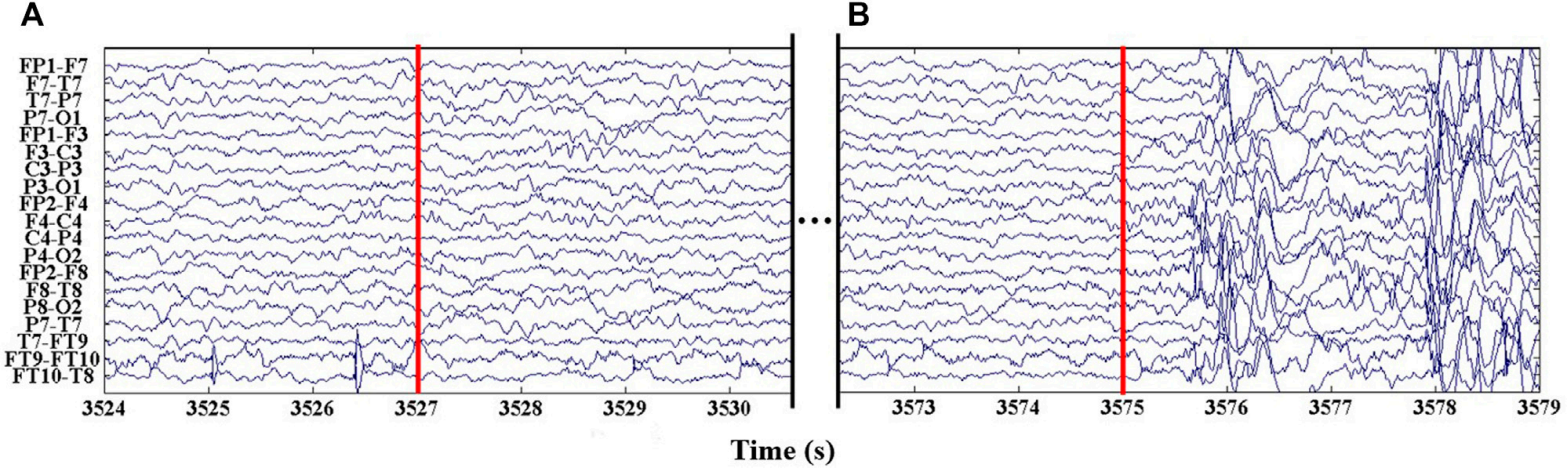
An epileptic segment of patient “Chb24.” **(A)** The marked epileptic seizure detected time is 3,527 s. **(B)** The actual time of epileptic seizure detected is 3,575 s.

The seizure detection latency for each seizure event is showed in Section 3.1.2. Benefiting from the calculations of multiple EEG channels by the persistent homology, the rhythmic changes of EEG signals can be captured sensitively, resulting in the good results with an average detection latency of 1.22 s. Notably, there was a rare high latency detection result in a seizure event in the subject “Chb24,” which seizure event is detection at a latency of 45 s. Figure 7 shows an EEG segment of the rare misclassification seizure event of “Chb24_11.” In Figure 7, the 3,257 s marked by red line **a** is the starting time of epilepsy, while the 3,575 s marked by red line **b** is the actual starting time of epilepsy detected in this study. It can be seen from the figure that in a period of time after 3,527 s, the EEG signal of the subjects remained stable, the rhythm did not change significantly, and there was no irregular abnormal waveform in each channel, which lasted until 3,575 s. After 3,575 s, the EEG signal began to change violently, the waveform was highly irregular, and the normal rhythm disappeared. It is the aforementioned discrepancy that the proposed approach classifies the segment 3,527–3,575 s as interictal, which is the reason for the large difference between the seizure detection time and the labeled seizure time for subject Chb24_11. Due to the lack of strict and unified epilepsy labeling standards, the physician’s labeling may have some errors with the actual seizure time; Moreover, the collection of EEG recordings is greatly affected by the environment, which impede the development of epilepsy detection. This is the main problem faced by the automatic epileptic seizure detection at present.

**FIGURE 8:**
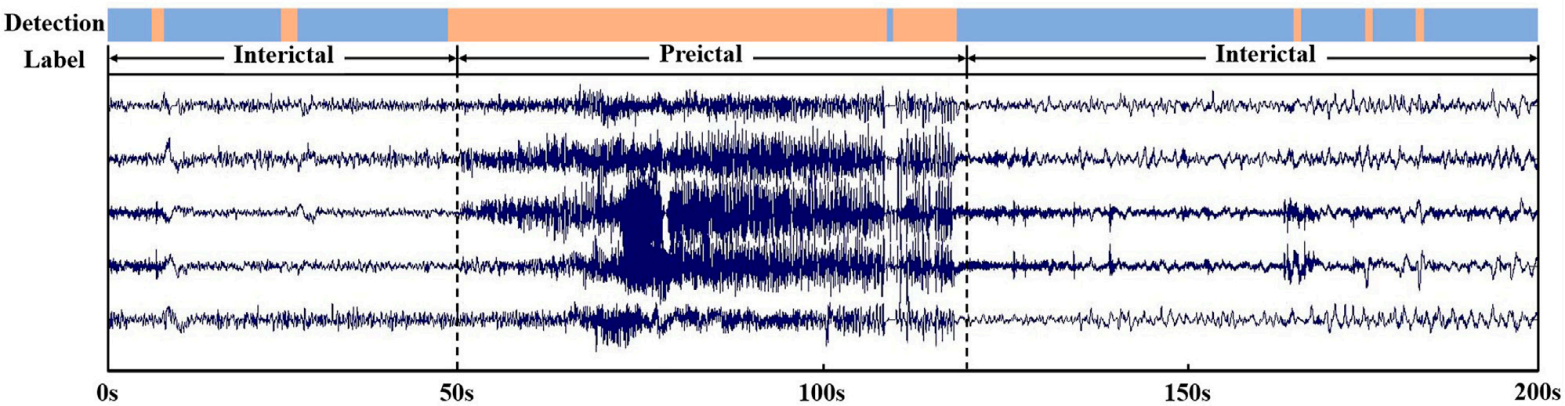
Detect an EEG signal with duration of 200s and containing seizure and interictal period.

For real-time signals performance analysis, we selected a segment of EEG signal including seizure and interictal period, the reliability of the epileptic seizure detecting method on real EEG signals was tested. As shown in the Figure 8, based on the original label, we know that epilepsy occurs at the 50 s and ends at the 120 s. And the proposed method detected epilepsy with a starting time of 49 s and an ending time of 119s. In addition, the detection results contain several errors that have a short duration of no more than 2 s, are all within acceptable error limits. The evaluation results show that the proposed method has capability to detect epileptic seizure.

### 4.5 Limitations and prospects

Owing to the different choices of channels during EEG acquisition, the different EEG databases cannot be unified, and even the channels of different subjects in the same database cannot be unified, resulting in the inability of joint use between databases, the inability to construct a large database, and the limited amount of epilepsy recording. Moreover, the quality of the EEG recording is greatly affected by the external disturbance and varies significantly between different subjects. These are the reasons that limit the development of automatic seizure detection research.

Although the great accuracy of epilepsy detection was obtained in this work, some improvements still need to be made. In addition, as a next step, we will study the multiple classification of EEG epilepsy species and the influence of the feature extraction. Another possible work is to apply persistent homology to the epilepsy prediction.

## 5 Conclusion

This study developed a novel automatic seizure detection approach based on the persistent homology to extract the topological features of high-dimensional EEG recordings. First to construct the Vietoris-Rips filtration model and then to generate the persistent homology barcode, and last applying GoogLeNet classifier to detect the seizure. The detection results obtained from the CHB-MIT Database recordings revealed that the proposed approach have great performance not only on segment-based experiment but also on an event-based experiment. Furthermore, the proposed approach was also evaluated using the Siena Scalp Database and the Bonn Database, both of which showed excellent results and all evaluation indexes are achieved over 95%. These results suggested that the proposed approach had high accuracy and robustness, as well as strong universality and applicability for automatic seizure detection. Moreover, this study shows that the proposed approach is not only applicable to binary classification of EEG, but also suitable for multiclassification. At the same time, we also explored the effects of VR complex filtration model construction on detection performance. Increasing the points in the point cloud can obtain more topological features, which could improve the detection quality, but the computation time also need to be considered. To summarize, this study shows that persistent homology has shown good performance in the detection of epilepsy. Persistent homology can directly process high-dimensional data without selecting channels and splicing features, which is quite suitable for multiple-channels EEG signals analysis. In addition, the method constructed in this paper provides a flexible application framework and lays a foundation for the analysis of EEG signals using persistent homology.

## Data Availability

Publicly available databases were analyzed in this study can be found here: CHB-MIT Scalp EEG Database: https://physionet.org/content/chbmit/1.0.0/, Siena Scalp Database: https://physionet.org/content/siena-scalp-eeg/1.0.0/ and Bonn Database: https://repositori.upf.edu/handle/10230/42894.
